# A review of recruitment of children to randomised clinical trials in the NIHR clinical research network portfolio

**DOI:** 10.1186/1745-6215-14-S1-P84

**Published:** 2013-11-29

**Authors:** Geetinder Kaur, Rosalind Smyth, Paula Williamson

**Affiliations:** 1 University of Liverpool, Liverpool, UK; 2 University College London (UCL), London, UK

## Background

Recruitment of children to randomised clinical trials is perceived to be difficult, although empirical evidence is lacking. We have undertaken a review of recruitment to paediatric trials in the National Institute of Health Research (NIHR) portfolio to understand the current situation and investigate factors that influence recruitment.

## Methods

Randomised clinical trials of any health care intervention, conducted between 2006 and 2012 and closed to recruitment, were identified. Recruitment performance was assessed by comparing achieved to planned recruitment. Putative factors affecting recruitment will be tested for association with successful recruitment, defined initially as recruitment to 100% of target in the planned time frame. Five factors were selected from an evidence based list of potential factors affecting recruitment; being an IMP (Investigational medicinal product) vs. non-IMP trial, trial of acute vs. chronic illness, CTU support, pilot or feasibility assessment and logistical burden of the trial as compared to routine care.

## Results

152 trials were identified. 52 trials did not meet the inclusion criteria and were excluded. 42% trials recruited successfully. 66% of the trials were IMP (Investigational medicinal product) trials and 34% were non-IMP trials. Preliminary analysis showed no statistically significant association between recruitment success and being an IMP trial (p=0.69). Full results will be reported.

## Conclusion

This study will provide a quantitative assessment of recruitment performance in paediatric trials and identify predictors of successful recruitment. This information will be very useful in developing strategies to counter the problem of under-recruitment in paediatric research.

